# Diagnostic challenge in isolated Neurosarcoidosis: A case report

**DOI:** 10.1016/j.radcr.2025.06.110

**Published:** 2025-07-29

**Authors:** Md. Deluwar Hussen, Zahin Shahriar, Zareen Tabassum

**Affiliations:** a Dinajpur Medical College, Dinajpur, Bangladesh; b Dhaka Medical College, Dhaka, Bangladesh

**Keywords:** Case report, Glucocorticoids, Magnetic Resonance Imaging (MRI), Neuro-sarcoidosis, Seizures

## Abstract

Neuro-sarcoidosis is one of the uncommon manifestations of sarcoidosis that presents with diagnostic difficulties, particularly when the patient does not show any signs of extra-neural disease. We report an 18-year-old female with an initial manifestation of neck pain, vomiting, seizures and cranial nerves palsies. The first MRI showed the T2 Flair hyperintensity, cortical T1 hyperintensity, nodular leptomeningeal enhancement and small focus of restricted diffusion, which is an isolated finding in the neuro-sarcoidosis. Peripheral nerve biopsy showed the presence of a non-caseating granuloma. This case brings out the challenges of diagnosing neuro-sarcoidosis due to the diverse forms of neurological manifestation of the disease and its mimicry of other diseases such as multiple sclerosis. Sarcoidosis manifestations do not have strong systematic features, and hence, requires sophisticated clinical, radiological and CSF analysis for a provisional diagnosis. This case points to the diagnostic significance of neuro-sarcoidosis in patients developing the neurological manifestations, as well as the necessity for the multiple-disciplinary approach to the management of the disease.

## Introduction

Sarcoidosis is an idiopathic symptomatic disease that is due to an overactive immune response to antigens in genetically predisposed individuals whose histological expression is non-caseating granuloma [1]. Antigens are processed by antigen processing cells and expressed to CD4+helper T cells which release proinflammatory cytokines causing activation of macrophages leading to the formation of granuloma [2].There are different stages in the development of sarcoidosis, the affected organs are mainly the lungs and lymph nodes [3]. Sarcoidosis affecting bones, dura mater, nerves, roots, leptomeninges and parenchyma can occur singly or in combination but the most common imaging abnormality involving head and spine corresponds to leptomeningeal enhancement, especially around the base anterior to the Sella turcica [4]. Lung is a site of involvement in almost 90% of the cases and is the major cause of morbidity and mortality related to this condition [5]. Other over-involved organs consist of skin (up to 30%); eye (10%-25%); and mediastinal and hilar lymph nodes (90%) [6]. Neurologic involvement in patients with sarcoidosis presents in the of meningitis with cranial nerve dysfunction to seizures or paralysis of extremities [7]. This is why neurologic complaints in patients with known sarcoidosis give a hint of the possibility of neuro-sarcoidosis [8]; nevertheless, such clinical presentation of sarcoidosis, isolated CNS manifestation without the presence of systemic symptoms, is very rare [9]. It has been estimated that 10% of patients with sarcoidosis has involvement of the central nervous system [10]; however, isolated neuro-sarcoidosis in the absence of other systemic manifestations is distinctly uncommon and occurs in somewhat less than 1 percent of patients with this disease [11]. Clinical symptoms of neuro-sarcoidosis are determined with the localization of the granulomatous inflammation and are generally the manifestations of the region of the brain affected [12]. Ophthalmoplegia – both the central or peripheral type of facial nerve palsies—and vision impairment are typical, along with other manifestations such as headache, seizures, and meningeal signs [13]. Several of these symptoms can be mimic signs or symptoms of multiple sclerosis like weakness, paresis, paresthesia, diplopia and dysarthria [14]. Spinal cord involvement may present as lower limb weakness and other non-specific myelopathic signs [15].

We faced several diagnostic challenges due to a low resource setting. The onset of the neurological symptoms in sarcoidosis usually follow systemic symptoms- but this patient presented with isolated neurological findings which is unusual and unique. Fascinatingly, this patient had presented with unilateral cranial nerve palsies which created more commotion. So, we think this case was very rare & valuable for physician to understand how neuro-sarcoidosis could have bizarre presentations. This case report has been written according to CARE guidelines.

## Case presentation

**Fig. 1:**
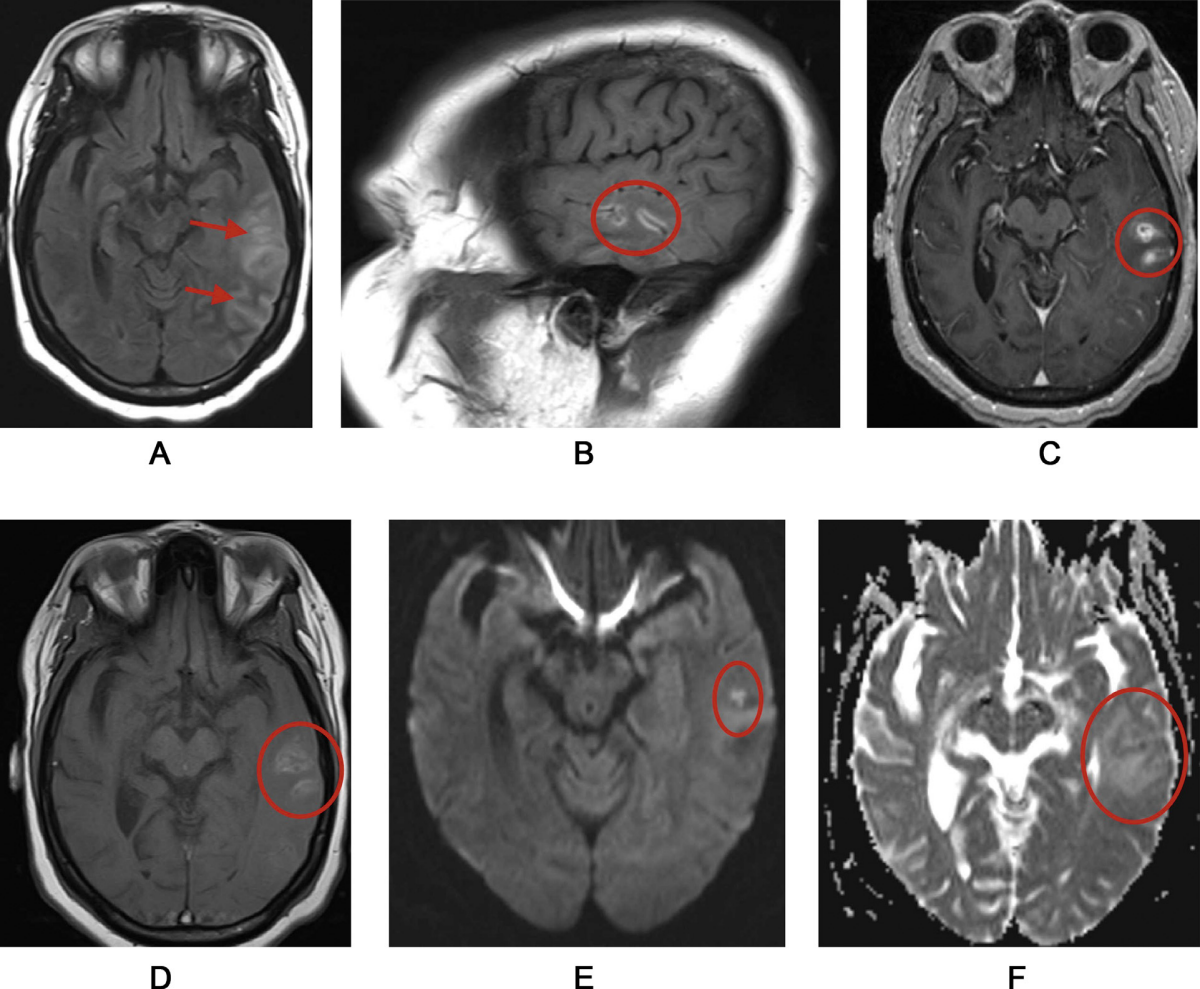
MRI reveals mildly expansile T2 FLAIR hyperintensity in the left temporal lobe, with cortical T1 hyperintensity, nodular leptomeningeal enhancement, and small foci of restricted diffusion. These indicate active inflammation, vasogenic edema, suspected cortical laminar necrosis, and cytotoxic edema of Neurosarcoidosis with associated vasculitis affecting the left temporal lobe.

A 18-year old female presented with a 3 month history of neck pain and vomiting. She also had seizures for one month and facial paralysis involving the lower right side. Soon after this, she had abdominal pain for two and a half months. There was no history of diabetes, asthma, hypertension, incarceration, travel or any exposure history. She had hospitalizations during the past several months as follows. One year prior to the presentation, she was admitted to a hospital with the symptoms of nasal regurgitation of food, dysphagia and dysarthria and had been diagnosed with bulbar palsy after cranial nerve assessment. Two months prior to this admission, she was complaining of food intolerance, back pain and neck pain; she was admitted with a diagnosis of meningoencephalitis. Initially, she was diagnosed to have an acute infarct in the periventricular, deep white matter, cortical and juxta-cortical areas which is a very uncommon manifestation of neuro-sarcoidosis (Fig. 1).

For several reasons this case is different from the typical case. First, the symptoms were neck pain and vomiting for 3 months following which the patient had developed more classical neurological symptoms in the form of seizures and facial nerve palsy. These early gastrointestinal symptoms, especially vomiting is not very common in neuro-sarcoidosis which commonly presents with cranial neuropathy or meningeal signs.

Further, the evolution of symptoms such as diplopia, hand tremors and watering of the right eye and their temporal distribution implies a multifocal neurological presentation. Neuro-sarcoidosis was diagnosed utilizing clinical data, imaging and CSF profile. In CSF analysis, proteins have increased as well as the glucose level and inflammatory pathology has been observed. Peripheral nerve biopsy showed non-caseating granuloma which also confirmed this diagnosis. Unfortunately, no other generalized sarcoidosis symptoms, including pulmonary involvement, were present, thus contributing to the difficulties with diagnosis. The neurological involvement was proximally, and hence neuro-sarcoidosis was established although there were no classical systemic symptoms. High-dose corticosteroids (IV methylprednisolone 1 gm/day for 5 days and then oral prednisone), levetiracetam for seizures and antibiotics (IV Ceftriaxone 2 gm twice daily) for meningoencephalitis were administered to the patient. Patient showed improvement in cranial nerve function and swallowing with the treatment. So, no other immunosuppressive drugs were added. However, neurological symptoms recurrence meant that she needed to continue with immunosuppressive therapy for neuro-sarcoidosis. Despite limited resources we took neurology consultation and did a sural nerve biopsy revealing noncaseating granuloma and acted based on clinical suspicion and CSF and other noninvasive investigations and imaging along with the biopsy finding.

## Discussion

In most cases, neurosarcoidosis presents with early signs in the nervous system like headache, dizziness, fatigue, visual disturbance, ataxia, weakness or numbness in one side, facial nerve palsy and involvement of brain and spinal cord tissue, along with common systemic signs such as enlarged lymph nodes near the lungs and skin sores [[16], [17], [18]]. Whereas, this patient first showed non-identical symptoms, having neck pain and being sick with vomiting for over 3 months. Later phases of the disease involved seizures and paralysis of the facial nerve muscles. Another interesting feature is the role of periventricular, deep white matter, cortical and juxta-cortical areas which were presumed as an isolated infarct. The usual locations of CNS involvement in neurological sarcoidosis are basal ganglia, Brainstem and meninges [19]. Rarely the periventricular, deep white matter, cortical and juxta-cortical areas have been found with lesions in neuro-sarcoidosis Table 1.

**Table 1:** Lab findings summary.

Test	Parameters	Findings	Reference range
Complete blood count	Hemoglobin (Hb)	11.8 g/dL	12–16 g/dL (female)
	White blood cell count (WBC)	9.01 × 10^6 /cumm	4.11 × 10^6 /cumm
	Neutrophils	80%	52-62%
	Platelet count	255 × 10^9 /L	150–400 × 10^9 /L
	ESR	27 mm in 1st hour	0–20 mm in 1st hour
Serum electrolytes	(Na/K/Cl)	134/4.18/99 mmol/L	Na: 136–148, K: 3.5–5.2, Cl: 96-108
CSF analysis	Leucocyte count	(≥ 5 cells/cumm)	0–5 cell/cumm
	Lymphocyte	87%	
	CSF Glucose	28.62 mg/dL	50–80mg/dl
	CSF Protein	53 mg/dL	15–40 mg/dl
	CD4+/CD8+ ratios	3.8	1-3
	CSF-ACE	6.1 U/L	0–2.8 U/L
	CSF-oligoclonal bands (OCBs)	Present	-
	IgG index	0.4	0.3–0.7
	Oligoclonal band	Absent	
	VDRL	Non-reactive	
Serum calcium		11.5 mg/dL	8.5–10.5 mg/dL
Serum ACE		95 U/L	8-65 U/L
VDRL	Non-reactive		
Nerve Biopsy		Indicative of Noncaseating Granuloma	
Serum PTH		12 pg/mL	10–65 pg/mL
Serum 1,25 Dihydroxy vitamin D		14 pg/mL	18–64 pg/mL (Male) 18–78 pg/mL (Female)

Differential diagnosis:

Neurosarcoidosis

Multiple sclerosis

Neurosyphilis

In this case, our major challenge in diagnosis was that this patient presented with neck pain, vomiting, and cranial nerve palsies all of which are not primary markers of neuro-sarcoidosis. The finding of periventricular, deep white matter, cortical and juxtacortical area involvement on MRI was quite rare, because neuro-sarcoidosis is usually characterized with enhancement of basal ganglia, brainstem and meninges. This specific imaging feature along with clinical symptoms made the diagnosis quite challenging and delayed identification of neuro-sarcoidosis as the source of the symptoms, CSF analysis revealed raised protein and low glucose but raised cell count with lymphocytosis, raised ACE, and presence of OCB; although suggestive of neuro-sarcoidosis, these are not specific enough to warrant a definite diagnosis, and thus added to the diagnostic challenge. Because of the lack of systemic sarcoidosis manifestations, clinical correlation and imaging findings guided care, underscore the role of a heightened suspicion for neuro-sarcoidosis when confronted with new acute neurological symptoms.

## Conclusion

The effectiveness of various diagnostic techniques in the management of atypical neuro-sarcoidosis is demonstrated in this case, raising clinical difficulties in the presentation of the disease. The fact that the diagnosis was made from seizures, periventricular, deep white matter, cortical and juxtacortical area lesions in the absence of other signs of sarcoidosis illustrates that the condition should always be considered in patients with neurological symptoms and that imaging studies are critical in the diagnosis of the disease. High-dose corticosteroids shows that good outcome can be expected once neuro-sarcoidosis is diagnosed early and treated, even with limited health care resources. This case should be of interest to clinicians and researchers at the interface of neurology- both as a cross-sectional survey of the wide manifestations neuro-sarcoidosis and as an affirmation of how multifaceted approaches to care should be when managing such complex neurological disorders.

## Author contributions

Md. Deluwar Hussen, MBBS (Dinajpur Medical College, Dinajpur): Dr. Deluwar was involved in the formulation of the study, as well as the attainment of the data collection and evaluation for the study. He also contributed to the writing of the review of literature and the manuscript and the review and editing of the manuscript. The clinical care of the patient during the period of this study was provided by Deluwar who besides being corresponding author of the article is supervising the whole team. Zahin Shahriar,MBBS, Dhaka Medical College, Dhaka: He has helped in manuscript writing, revision, data curation. Zareen Tabassum, MBBS, Dinajpur Medical college, Dinajpur: She completed a final entire typed manuscript and offered contribution on how the manuscript should be reviewed for content or ideas. She also took time to refine the manuscript. and also assisted in the preparation of review of literature and writing of the manuscript and reviewing and editing of the manuscript.

## Data sharing statement

As for the accessibility of the data of this study, the datasets are available from the corresponding author on reasonable request. In view of the case report, informed consent to use identifiable data was sought from the patient and in the exceptional circumstance that data is to be disclosed it will only be done after the patient’s details are anonymised. Users’ request should be combined with a declaration of the reasons why such request is made as well as a declaration of how the data is to be utilized. The data will be spread out in compliance with all the ethical scenarios from the study and the corresponding institutional guidelines.

## Ethics statement and consent for publication

The authors declare that written informed consent was obtained for the publication of this manuscript and accompanying images using the consent form provided by the Journal.

Generative AI use

For paraphrasing of citations and basic structure of outline, actually very limited use of Quillbot has been made and all have been edited properly after review.

## Approval of the research protocol

N/A.

## Informed consent

All informed consent was obtained from the subject and from all authors for publication of this paper.

## Registry and the registration No. of the study/trial

N/A.

## Animal studies

N/A.

## Data sharing statement

As for the accessibility of the data of this study, the datasets are available from the corresponding author on reasonable request. In view of the case report, informed consent to use identifiable data was sought from the patient and in the exceptional circumstance that data is to be disclosed it will only be done after the patient’s details are anonymised. Users’ request should be combined with a declaration of the reasons why such request is made as well as a declaration of how the data is to be utilized. The data will be spread out in compliance with all the ethical scenarios from the study and the corresponding institutional guidelines.

## Patient consent

All informed consent was obtained from the subject.

## Appendix Supplementary materials

Image, application 1
