# Supplementary material for: Diagnostic challenge in isolated Neurosarcoidosis: A case report
Source: Radiol Case Rep. 2025 Jul 29;20(10):5233–8. doi: 10.1016/j.radcr.2025.06.110 (PMC12332201; doi:10.1016/j.radcr.2025.06.110)
Supplement: Supplementary file 1 [file mmc1.docx]

**Funding information:**

Although this research is mostly theoretical, no funding was specifically provided by any agent in the public, commercial or not for profit sectors.

**Data sharing statement:** As for the accessibility of the data of this study, the datasets are available from the corresponding author on reasonable request. In view of the case report, informed consent to use identifiable data was sought from the patient and in the exceptional circumstance that data is to be disclosed it will only be done after the patient’s details are anonymised. Users’ request should be combined with a declaration of the reasons why such request is made as well as a declaration of how the data is to be utilized. The data will be spread out in compliance with all the ethical scenarios from the study and the corresponding institutional guidelines.

**Ethics statement and consent for publication:**

The authors declare that written informed consent was obtained for the publication of this manuscript and accompanying images using the consent form provided by the Journal.

**Generative AI Use:** For paraphrasing of citations and basic structure of outline, actually very limited use of Quillbot has been made and all have been edited properly after review.
